# Calcium-Dependent Interaction of Nitric Oxide Synthase with Cytochrome *c* Oxidase: Implications for Brain Bioenergetics

**DOI:** 10.3390/brainsci13111534

**Published:** 2023-10-31

**Authors:** Virginia Haynes, Cecilia Giulivi

**Affiliations:** 1 School of Veterinary Medicine, Department Molecular Biosciences, University of California Davis, Davis, CA 95616, USA; 2 Medical Investigation of Neurodevelopmental Disorders (MIND) Institute UCDH, University of California Davis, Sacramento, CA 95817, USA

**Keywords:** mitochondria, nitric oxide, nitric oxide synthase, protein–protein interaction, Complex IV, bioenergetics, oxygen

## Abstract

Targeted nitric oxide production is relevant for maintaining cellular energy production, protecting against oxidative stress, regulating cell death, and promoting neuroprotection. This study aimed to characterize the putative interaction of nitric-oxide synthase with mitochondrial proteins. The primary finding of this study is that cytochrome *c* oxidase (CCO) subunit IV (CCOIV) is associated directly with NOS in brain mitochondria when calcium ions are present. The matrix side of CCOIV binds to the *N*-terminus of NOS, supported by the abrogation of the binding by antibodies towards the *N*-terminus of NOS. Evidence supporting the interaction between CCOIV and NOS was provided by the coimmunoprecipitation of NOS from detergent-solubilized whole rat brain mitochondria with antibodies to CCOIV and the coimmunoprecipitation of CCOIV from crude brain NOS preparations using antibodies to NOS. The CCOIV domain that interacts with NOS was identified using a series of overlapping peptides derived from the primary sequence of CCOIV. As calcium ions not only activate NOS, but also facilitate the docking of NOS to CCOIV, this study points to a dynamic mechanism of controlling the bioenergetics by calcium changes, thereby adapting bioenergetics to cellular demands.

## 1. Introduction

Gasotransmitters refer to a class of gases produced by the human body, which can diffuse freely between membranes and act as signaling molecules, critical in regulating various physiological processes [1]. These molecules play crucial roles in the regulation of signaling and intracellular homeostasis. The ones identified so far include carbon monoxide (CO), nitric oxide (NO^•^), and hydrogen sulfide (H_2_S). NO^•^ is generated enzymatically in vivo by nitric-oxide synthase (NOS) during the conversion of L-Arg to citrulline. NO^•^ is a major endogenous mediator involved in many pathophysiological functions such as blood pressure regulation, inhibition of platelet aggregation, and neurotransmission [2,3,4,5]. Its versatile nature also extends to cellular signaling, where it participates in processes such as inflammation and the regulation of cellular metabolism. NO^•^’s multifaceted role in the body underscores its significance as a gasotransmitter, showcasing the intricate ways small molecules like NO^•^ can profoundly impact human health and physiology.

Three isoforms of NOS are expressed in mammals and differ in their functions, amino acid sequence, post-translational modification, and cellular location [4]. Two NOS, neuronal NOS (NOS1) and endothelial NOS (eNOS or NOS3), are constitutively expressed and involved in signal cascades. The third NOS is cytokine-inducible (iNOS or NOS2) and functions as both a regulator and effector of the immune response. All three NOS isoforms were recovered from primary cells and the brain in soluble and particulate fractions [6]. The advantage of this subcellular location diversity, function, and regulation confers high controls for localized NO^•^ production. For example, NOSs differ significantly regarding calcium ions levels required to bind calmodulin [4,7] and may respond to S/T phosphorylation [7,8]. NOS1 and NOS3 rely on both calcium ions and calmodulin for activation, with calcium ions binding to calmodulin being a critical step in their activation. In contrast, NOS2 is typically expressed in response to inflammatory signals and calmodulin is tightly bound to it, thereby becoming activated when any calcium ions are present in the nearby milieu. These distinct requirements for calcium and calmodulin reflect the specialized roles of these NOS isoforms in physiological and pathological processes within the body.

Our studies were the first to provide direct evidence for NO^•^ production by purified rat liver mitochondria [9,10,11], by an NOS mainly localized at mitochondrial membranes [12]. Purified NOS from rat liver is the alpha NOS1 isoform, constitutively expressed, and membrane-bound, with a different acylation pattern from eNOS [13]. Other studies using cultured cells with a CMV-promoter to overexpress eNOS located this protein at the outer mitochondrial membrane through a five amino acid long docking sequence [14].

Importantly, depending on the tissue and condition investigated, all three NOS isoforms seem to be temporarily associated with mitochondria. Even if NO^•^ is a small, free diffusing molecule, identifying an NOS that colocalizes with mitochondria has established the basis for a reinterpretation of a localized regulation by NO^•^ of mitochondrial energy metabolism, oxygen consumption, and oxygen-free radical production [12,15]. Also relevant, NO^•^ participates in mitochondrial fusion and fission processes, ensuring a balanced and functional mitochondrial network [16]. This dynamic balance is crucial for adapting to changing energy demands and maintaining cellular health.

We showed that the production of NO^•^ by mitochondria modulated the oxygen consumption of the organelle by competitive inhibition of Complex IV or cytochrome *c* oxidase (CCO) [17,18]; and, consequently, the oxygen free-radical production [18]. Inhibition of oxygen consumption is transient if small quantities of NO^•^ are generated. However, high or sustained levels of NO^•^ inhibit the respiratory chain at the CCO step, damaging either electron respiratory chain components other than CCO or key players of the mitochondrial dynamics [16,19,20], leading to an enhanced rate of oxygen free radicals that, in turn, may damage mitochondria [18] and/or initiate the release of cytochrome *c* ensuing in apoptosis [21].

Considering the critical role that NO^•^ may have in regulating the oxygen consumption by the cell and the oxygen distribution [22], we investigated the protein–protein interactions of brain NOS with mitochondrial proteins and under conditions at which NOS becomes activated. Experiments provided by Persichini et al. [23] suggested a direct interaction of CCO subunit Va (CCOVa) with NOS. This interaction was interpreted to occur via binding the *C*-terminus end of CCOVa (which contains a class III *C*-terminal peptide as the ligand) to the PDZ domain of NOS. However, analyses of CCO subunits indicate that the *C*-terminus of Va is not free but interacts directly with CCO (especially subunit II), no record of such interaction is found under BioGrid or IntAct the major databases on protein–protein interaction (Supplementary File S1), and that CCOVa is located in the cytosolic domain of the oxidase Complex [24], thus prompting a reconsideration of the direct binding of any protein to the Va *C*-end as originally proposed. In addition, these previous studies did not provide information on the putative role of calcium ions for the NOS–CCO interaction, given that the activation of constitutive NOSs requires calcium ions and calmodulin, and the presence of these cofactors may affect the protein–protein interactions.

Reevaluating this interaction, our results show that NOS interacts with CCOIV in a calcium-dependent manner. The interaction with CCOVa could be envisioned after this primary interaction. This is supported by various approaches, from proteomics to far Western blotting techniques. Understanding this association in detail is important because it may profoundly impact the regulation of energy balance in the brain.

## 2. Materials and Methods

Biochemicals and chemicals—All reagents were of analytical grade or higher. Fatty-acid free bovine serum albumin was purchased from ICN (Irvine, CA, USA). Purified mouse IgG2a was purchased from ICN Biomedicals, Inc. (Aurora, OH, USA). Purified mouse IgG, mouse monoclonal antibodies to NOS1 clone B1, calmodulin, percoll, protease inhibitor cocktail, protein G immobilized on agarose beads, beta-mercaptoethanol, Sigmascreen streptavidin coated 96-weel plates, and PMSF were purchased from Sigma (St. Louis, MO, USA). Protease inhibitor cocktail set III was purchased from Calbiochem (San Diego, CA, USA). Tris, glycine, sodium chloride, potassium chloride, D-mannitol, sucrose, and EDTA were purchased from Fisher (Pittsburg, PA, USA). The concentrations of antibodies or their dilutions used in this study are indicated in the text. Original trials included using the concentrations recommended by the manufacturer, but on some occasions, they were adjusted to optimize the signal-to-noise ratio. Rabbit polyclonal anti-NOS1 (R20) and the normal rabbit IgG agarose conjugate were purchased from Santa Cruz Biotechnology Inc. (Santa Cruz, CA, USA). Goat anti-mouse HRP conjugate was purchased from Upstate Biotechnology (Lake Placid, NY, USA). Goat anti-rabbit HRP conjugate was purchased from BD Transduction Biosciences (San Diego, CA, USA). Mouse monoclonal anti-OXPHOS CCO subunit I (clone 1D6) and subunit IV (clone 20E8C12) antibodies were purchased from Molecular Probes (Portland, OR, USA). Recombinant NOS1 was purchased from Cayman Chemical. Supersignal ELISA Femto Maximum Sensitivity Substrate, ProFound Pull-down Biotinylated Protein:protein interaction kit, BCA protein assay kit, and the EZ-link sulfo-NHS-LC biotinylation kit were purchased from Pierce Biotechnology (Rockford, IL, USA). Rainbow protein markers, PhastSystem Gels, and SDS buffer strips were purchased from Amersham (Piscataway, NJ, USA).

Isolation of rat brain mitochondria and inner membrane fractions—The experimental protocol adhered to the Guide for the Care and Use of Laboratory Animals (NRC 1985) and was approved by the Institutional Animal Care and Use Committee (protocol number 21780) of the University of California-Davis. Adult male Wistar rats (176–200 g) were obtained from Charles River (Wilmington, MA, USA). This study was fully aligned with the ARRIVE 2.0 guidelines [25], ensuring transparent and comprehensive reporting of the research methods utilized in this study and its outcomes. Adhering to these guidelines enhances research quality and reproducibility, underscoring our commitment to upholding rigorous standards and promoting transparency in our findings. We confirm that all experiments were performed following relevant guidelines and regulations. All experimental procedures were strictly performed per the ethical requirements of IACUC at the University of California Davis.

Brain mitochondria were isolated as previously described by our laboratory [26,27]. Briefly, purified mitochondria from homogenized cortex were collected by differential centrifugation followed by Percoll gradient and multiple washes in MSHE buffer (220 mM D-Mannitol, 70 mM sucrose, 0.5 mM EGTA, 2 mM HEPES, 0.1% fatty-acid free bovine serum albumin) and 150 mM KCl. The mitochondria were used fresh or frozen at −80 °C in MSHE buffer containing protease inhibitor cocktail 1 µL/55 mg protein (Calbiochem set III supplemented with 10 mM EDTA). The inner membrane was purified with a discontinuous sucrose gradient [28,29]. Selected marker enzymes were tested to evaluate the purity of the mitochondrial subfractions: Assay for monoamine oxidase—Monoamine oxidase activity was measured spectrofluorometrically (315 and 410 nm, excitation and emission wavelengths, respectively) by coupling the production of H_2_O_2_ with the oxidation of *p*-hydroxyphenylacetic acid catalyzed by horseradish peroxidase. The reaction mixture contained 40 μM *p*-hydroxyphenylacetic acid, 5 U/mL horseradish peroxidase, 10 mM benzylamine, and 5–150 μg/mL mitochondrial protein in 70 mM potassium phosphate buffer, pH 7.2. Assay for adenylate kinase activity—Adenylate kinase activity was determined spectrophotometrically at 340 nm by coupling the production of ADP to the oxidation of NADH via the reactions catalyzed by pyruvate kinase and lactic dehydrogenase. The reaction mixture contained 0.4 mM NADPH, 0.1 mM ATP, 0.1 mM phosphoenolpyruvate, 1 μM rotenone, 0.04 U/mL of each pyruvate kinase and lactate dehydrogenase, 0.1 mM AMP, 0.04% Lubrol, 6 mM MgCl_2_, 130 mM KCl, 0.1 M Tris·HCl (pH 7.5), and 20–400 μg/mL of mitochondrial protein. Assay for cytochrome oxidase activity—Cytochrome oxidase activity was determined by spectrophotometrically measuring the oxidation of 50 μM ferrocytochrome *c* at 550 nm in a reaction mixture containing 0.25 M sucrose, 5 mM MgCl_2_, and 50 mM HEPES (pH 7.4), with 10–70 μg/mL of mitochondrial protein. Assay for malate dehydrogenase activity—This activity was determined by spectrophotometrically measuring the formation of NADH at 340 nm. The reaction mixture contained 0.5 mM NADH, 0.25 mM oxaloacetate, 4 μM rotenone, 80 μM Na_2_S, and 10–200 μg/mL mitochondrial protein in 25 mM potassium phosphate (pH 7.4). Assay for superoxide dismutases—Superoxide dismutase activities were assessed at pH 7.8 and pH 10.0 by evaluating the inhibition of cytochrome *c* reduction by superoxide anion, generated by xanthine plus xanthine oxidase [30] in the presence of 10 µM KCN (to eliminate the putative interference of cytochrome *c* oxidase activity, and this concentration has no effect on the cyanide-sensitive CuZn SOD and xanthine oxidase activities) [31]. Since rat CuZn SOD is 6–9 times more active at pH 10.0 than at pH 7.8, whereas Mn SOD purified from rat tissue [30] has the same activity at both pHs, the measurement of SOD activity at each pH permitted the calculation of the relative contribution of each isoform to the total SOD activity.

Inner membrane fractions were supplemented with 10–15% glycerol and proteolytic inhibitors and stored at –80 °C until use and then thawed on ice. Protein was determined by the Lowry assay using bovine serum bovine albumin as a standard [32].

Bioenergetics evaluation—Oxygen uptake linked to ATP production by isolated mitochondria (usually 0.2 to 0.5 mg protein/mL) was determined by polarography at 20–22 °C using a Clark-type oxygen electrode as described previously [33]. Briefly, samples were added to buffer containing 100 mM KCI, 75 mM mannitol, 25 mM sucrose, 1 mg/mL fatty-acid free bovine serum albumin, 5 mM potassium phosphate, 0.05 mM KEDTA, and 10 mM HEPES (pH 7.4) in a final volume of 0.2 to 1 mL. Incubation conditions for determining respiratory control ratios were at State 3 respiration (0.3 mM ADP, 10 mM succinate, 0.1 µg/mL rotenone) and State 4 respiration (after ADP was depleted) as defined by Chance and Williams [34]. Five paired experiments were run with and without the indicated calcium ions addition. For the evaluation of NO^•^ production on State 3 respiration, mitochondria (0.5 to 1 mg protein/mL) were incubated in 125 mM KCl, 1 mM MgCl_2_, 5 mM potassium phosphate, 1 mM HEDTA, 0.2 mM EGTA, 50 mM MOPS, pH 7.4, with 10 mM alpha-ketoglutarate, 0.3 mM ADP, and 4–6 nmol calcium chloride/g protein where indicated. The media were supplemented with 1 mM L-Arg or NMMA [35].

NOS preparation from brain mitochondrial membranes and purified NOS—Rat cortex from 5 animals were combined, and mitochondria were isolated as previously described [11]. The purified mitochondria were homogenized in Buffer A (1 mM EDTA, 5 mM 2-mercaptoethanol, 100 µg/mL protease inhibitor cocktail III (Calbiochem, San Diego, CA, USA), 1 mM PMSF, and 50 mM HEPES, pH 7.5). The homogenate was centrifuged for 1 h, 4 °C, at 150,000× *g*. The supernatant was decanted, and the pellet was homogenized in Buffer B (Buffer A plus 1 M KCl and 10% glycerol). The homogenate was centrifuged 150,000× *g* for 30 min at 4 °C. The supernatant was decanted, and the pellet was homogenized with Buffer A containing 20 mM CHAPS. The homogenate was kept on ice for 30 min, homogenized again, and centrifuged for 30 min at 150,000× *g*, 4 °C [11]. This fraction was called “crude NOS”, whereas purified NOS was obtained from this crude preparation as described before [11]. Protein content was determined for the supernatant, and Western blots were performed to confirm the presence of nitric oxide synthase.

Immunoprecipitation of NOS with *C*-terminus antibody for 2D-gel electrophoresis. Freshly isolated, purified mitochondria were diluted in ice-cold immunoprecipitation (IP) buffer (150 mM NaCl, 5 mM EDTA, 1 mM PMSF, 0.1% Triton X-100, 1 µL Protease Inhibitor Cocktail/20 mg protein, and 10 mM HEPES [36] to 2 mg/mL and lysed by addition of 1 mg digitonin *per* 10 mg mitochondrial protein. Where indicated, the lysis buffer also contained either 4 nmol of calcium ions/mg of protein or 20–25 mM EGTA. Mitochondria were incubated for 1 h on ice and then cleared by centrifugation at 14,000× *g* for 4 min. The rabbit polyclonal anti-NOS (40 µg) was prebound to protein-G agarose beads for 30 min at 20–22 °C in IP buffer. Beads were blocked with IP buffer containing 2% bovine serum albumin overnight at 4 °C, and the excess of albumin or antibodies was removed by washing the beads 5 times with IP buffer. Mitochondrial protein (2 mg) in solution was combined with the prebound beads (1:50 antibody:protein) and rotated for 1 h at 4 °C. Alternatively, normal rabbit IgG prebound to agarose beads was washed in IP buffer and used in place of the anti-NOS antibody (mock). The beads were washed 5 times in IP buffer without digitonin to remove the nonspecific binding. Twenty µL aliquots of beads were diluted with 20 µL 2× Laemmli buffer and heated to 95 °C for 5 min for the next separation step.

Two-dimensional electrophoresis of NOS IP and MALDI-TOF MS analysis—Beads collected and washed from the standard immunoprecipitation method described in detail above were incubated for 1 h at 20–22 °C with rehydration buffer (6 M urea, 2 M thiourea, 2% Nonidet P-40, 2% ampholytes, 0.1 M dithiothreitol) and spun in prepackaged columns (Pierce Biotechnology; Rockford, IL, USA) to remove beads from the denatured proteins/rehydration buffer. Two-dimensional electrophoresis (2-D electrophoresis) separates proteins according to two independent properties in two discrete steps: the first-dimension step, isoelectric focusing (IEF), separates proteins according to their isoelectric points (pI); the second-dimension step, SDS-polyacrylamide gel electrophoresis (SDS-PAGE), separates proteins according to their molecular weight. First dimension: The rehydration buffer containing the denatured proteins was used to rehydrate IEF strips (Amersham) overnight at 20–22 °C in an Immobiline DryStrip reswelling tray. The IEF was performed using the Multiphor II apparatus (Amersham Pharmacia Biotech, Piscataway, NJ, USA). Following the first-dimension run, the strips were equilibrated for the second dimension for 10 min in 0.05 M Tris, pH 6.8, 6 M urea, 30% glycerol, 1% SDS, and 25 mg/mL of dithiothreitol. Second dimension: After equilibration, the IPG strips were laid atop a 15% SDS-PAGE gel and layered with 0.5% agarose in 1× Laemmli running buffer. The electrophoresis was performed using the Hoefer SE 600 at 50 V overnight at 4 °C. After electrophoresis, gels were stained with SYPRO Ruby™ (Molecular Probes), and spots not appearing in the mock sample were picked and digested for MALDI-TOF MS with sequencing-grade trypsin (Promega; Madison, WI, USA). The samples were promptly excised for subsequent processing to reduce UV exposure and enhance protein extraction.

Consequently, images were not captured, and this task would be undertaken at a separate facility. Eluted peptides were analyzed at the University of Minnesota Mass Spectrometry Facilities (Minneapolis, MN, USA) as previously described [13]. The mass fragments were analyzed using two programs based on different algorithms: MS-Fit (ProteinProspector, version 3.4.1.) and ProFound (version 4.10.5).

**Figure 1:**
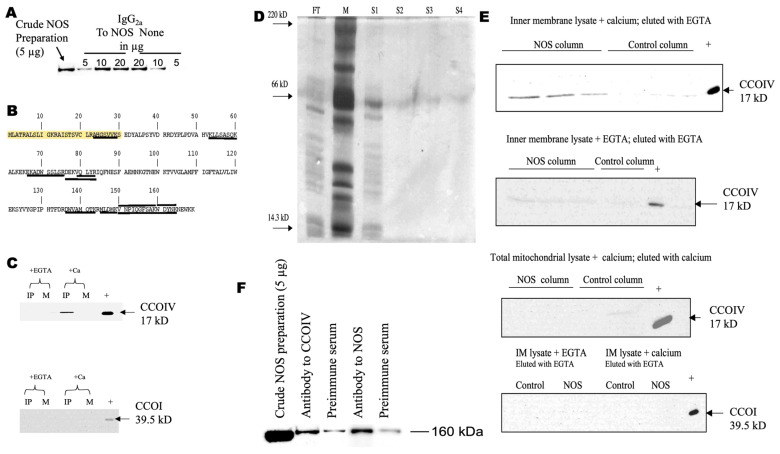
Brain NOS interacts with CCOIV. Panel (**A**) shows immunoprecipitation of NOS1 from a crude brain mitochondrial preparation with increasing amounts of antiNOS1 antibody directed to *N*-terminus of NOS1 (Sigma). Mock IPs were performed with matched protein amounts of preimmune mouse IgG_1_ in HEPES buffer with CHAPS (labeled as None). Western blots were performed with the solubilized proteins after extensive washes and probed with antibodies to NOS1 against its *C*-terminus (Santa Cruz). The positive control is 5 µg of crude NOS preparation. See all details under Methods. Panel (**B**) shows the primary sequence of CCOIV. Underlined sequences correspond to the mass tryptic digest obtained by MALDI-TOF MS. Highlighted, target mitochondrial sequence. In Panel (**C**), brain mitochondrial lysate was supplemented with calcium ions (+Ca) or EGTA (+EGTA) and incubated with rabbit polyclonal anti-NOS1 or preimmune rabbit IgG, both antibodies prebound to agarose beads. Immunoprecipitated proteins were separated on a 12.5% SDS-PAGE, transferred to PVDF, and then probed with (top panel) anti-CCOIV antibody (17 kD) and (bottom panel) anti-CCOI antibody (57 kD theoretical and 39.5 kD experimental on SDS-PAGE). IP, immunoprecipitation with calcium ions or EGTA; M, mock IP with rabbit IgG conjugated beads; +, positive control, 5 μg of brain mitochondrial protein lysate. In Panels (**D**,**E**), columns were prepared with recombinant NOS as bait (NOS column) or no bait protein and blocked with biotin (Control column). The columns were treated with mitochondrial brain inner membrane protein lysate. The flow-through was collected (FT), the column was washed four times with sodium acetate buffer containing 1 M NaCl, and each wash was collected (S1 through S4). Four μL of each flow-through (diluted 1:10) and wash (undiluted) was loaded per lane and separated in a 12.5% SDS-PAGE on a PhastSystem electrophoresis unit, then stained with silver staining protocol (following Amersham Pharmacia manual; M, molecular weight markers; Panel (**D**)) or transferred to PVDF membranes for Western blots (Panel (**E**)). Within Panel (**E**), the top panel probed for CCOIV: Lanes 1–3, NOS column incubated with brain mitochondrial inner membrane lysate containing calcium ions and eluted with EGTA 70, 60, and 50 μL, and lanes 4–6 control columns, 70, 60, and 50 μL. Lane 7, positive control with 2 μg of inner membrane lysate. Second from top probed for CCOIV: Lanes 1–3, NOS column with inner membrane lysate with EGTA and eluted with EGTA, loaded as for A. Control column lane 4, 70 μL, lane 5, positive control. Third from top probed for CCOIV: total mitochondrial lysate with calcium ions and eluted with calcium ions, positive control 5 μg whole mitochondrial lysate. Fourth from top probed for CCOI: Inner membrane lysate with calcium ions and eluted with EGTA (right) or EGTA and eluted with EGTA (left); 80 µL per lane, positive control 5 μg inner membrane lysate. Panel (**F**) shows 2 µg of anti-CCOIV was prebound to protein-G agarose beads and blocked as previously described. Two µg of mouse IgG_2a_ was used for mock IP. Crude NOS (1 µg/µL) in HEPES buffer with CHAPS was incubated with the beads for 1 h, rotating in an orbital shaker at 4 °C. Beads were washed 3× with HEPES buffer with CHAPS and 2× with HEPES buffer without CHAPS. Complexes were removed from beads by heating to 95 °C for 5 min in 2× Laemmli buffer. Positive control for IP of NOS was 10 µg of anti-NOS1 (*N*-terminus) or 10 µg of mouse IgG1 (mock). Positive antibody control was 5 µg of crude NOS preparation. Solubilized proteins were separated on 7.5% SDS-PAGE. Western blots for NOS were performed using the *C*-terminal anti-NOS1 antibody.

Immunoprecipitation of NOS by *N*-terminus directed antibody. Ten µL of washed protein-G agarose beads (Sigma) were each prebound with 5, 10, or 20 µg of anti-NOS1 antibody (*N*-terminus epitope; Sigma) for 30 min at 20–22 °C in an orbital shaker. The beads were blocked with 2% bovine serum albumin in HEPES buffer, then washed five times with HEPES buffer and incubated with 500 µg of crude (freshly prepared) brain mitochondrial NOS preparation in HEPES buffer with CHAPS (50 mM HEPES, 1 mM EDTA, 20 mM CHAPS, pH 7.5). Mock IPs were performed with matched protein amounts of preimmune mouse IgG_1_ in HEPES buffer with CHAPS. NOS–antibody–bead complexes were collected after 1 h in an orbital shaker at 4 °C. The beads were washed three times with HEPES buffer with CHAPS and twice with HEPES buffer without CHAPS. The beads were incubated in 2× Laemmli buffer for 5 min at 95 °C. Solubilized proteins were separated on 7.5% SDS-PAGE, and Western blots were performed using anti-NOS1 (*C*-terminal; Santa Cruz Biotechnology) to detect NOS bands collected by the *N*-terminus antibody. Positive controls included 5 µg of crude NOS preparation. Bands were visualized on a Kodak IS 1000 (depicted in Figure 1A). Once the optimal amount of antibody was identified, then 10 μg of anti-NOS1 was prebound to 10 μL of washed beads; the beads were incubated with 500 μg of crude NOS without calcium ions, with calcium ions, and 20 μg/mL calmodulin or with 20 mM EGTA in the CHAPS buffer; mouse IgG was used for the mock experiment.

Immunoprecipitation of CCOIV. This immunoprecipitation was performed by using a crude, freshly prepared sample of NOS. To this end, 2 μg of antibody to CCOIV was prebound to washed beads and incubated with 200 μg of crude NOS protein. Mouse IgG_2a k_ was used for the mock experiment. Washes and blocking were performed as described under immunoprecipitation of NOS with C-terminus antibody for 2D-gel electrophoresis.

Western blot for NOS, CCOI, and CCOIV—The supernatant containing proteins eluted from the agarose beads (above) or from Pull-down columns (below) were separated by SDS-PAGE (12.5% or 7.5%). Gels (12.5%) were transferred to PVDF for 24 min at 10 V in a semidry apparatus using buffer containing 20% methanol for proteins < 66 kD. For proteins greater than 66 kD, 12.5% gels were transferred to PVDF for 1 h 20 min at 15 V in a buffer containing 5% methanol. The 7.5% gels were transferred at 1.2 mA/cm^2^ blot for 1 h and 15 min. All membranes were blocked for 1 h at 20–22 °C in 5% nonfat dry milk. Anti-NOS1 antibody (Santa Cruz) at 1:500 was used to probe for NOS. Goat anti-rabbit-HRP (BD transduction labs) followed by ECL reagent was used to detect the bands. Anti-CCO antibodies were used at 0.3 µg/mL and 0.35 µg/mL to probe for subunits I and IV, respectively. Goat anti-mouse-HRP (Upstate) followed by ECL was used to detect bands.

Biotinylation of recombinant NOS—Recombinant NOS preparation (Cayman Chemical Co., Ann Arbor, MI, USA) was dialyzed against PBS at 4 °C overnight and then centrifuged in a 100 K filter (Millipore, Burlington, MA, USA) at 2000× *g* for 20 min. The dialyzed and filtered NOS was biotinylated with Sulfo-NHS-LC biotin per Pierce Biotechnology protocol and dialyzed against PBS overnight 4 °C to remove unreacted biotin. Protein determination of the dialyzed biotinylated protein was determined by Bradford protein assay. SDS-PAGE stained with Coomassie was used to confirm the concentration of recombinant NOS before column preparation.

Streptavidin/Biotin column preparation—Pull-down columns were prepared as per Pierce protocol. Briefly, 150 µg biotinylated NOS or no protein (TBS only) was bound to prewashed immobilized avidin-linked beads for 2 h, rotating at 4 °C. The column was washed, blocked with additional biotin, and washed again with TBS. Lysates were prepared as above. One mg of total protein from each lysate was incubated with either the NOS-bound column or a control column containing only biotin and avidin for 1 h, rotating at 4 °C. Flow-through was collected and followed by four washes with sodium acetate wash buffer (pH 5.0) provided in the kit supplemented with 1 M NaCl. The columns were then eluted with elution buffer provided in the kit (pH 2.8) or elution buffer supplemented with 5 mM EGTA (pH 2.8). The elution buffer was neutralized with 1 M Tris pH 9.5. The proteins in the eluate were denatured with 5× Laemmli buffer (at 1:5) at 95 °C for 5 min, and Western blots were performed as above.

Peptide library—An overlapping set of biotinylated peptides representing the mature CCOIV were prepared based on our design by Mimotopes (Clayton Victoria, Australia). The lyophilized peptides were diluted following a protocol supplied by Mimotopes to a final dilution of 2.9 mg/mL. Working dilutions of peptides were made from this stock.

Far Western blotting—Ten μg of semipurified NOS or 1 μg of recombinant NOS was separated by 7.5% SDS-PAGE and transferred to PVDF as for Western blot. The PVDF was blocked for 1 h in 2% BSA/PBS and then cut into strips. Each strip was incubated with a different peptide at 1.5 g/mL concentration in 2% BSA/PBS for 1 h, at 20–22 °C. The strips were washed 6 times for 5 min each with PBS-Tween-20 0.05%, then incubated with avidin-HRP (Pierce) at 1:10,000 dilution of 0.1 μg/μL stock in 2% BSA/PBS for 1 h, at 20–22 °C. The strips were again washed 6 times 5 min each with PBS-Tween, and bands were detected with Supersignal West Femto (Pierce) and visualized on the Kodak IS 1000.

ELISA methodology—Peptide 12 was diluted to ~10 µg/mL in PBS and loaded, in duplicate, into a 96-well plate prebound with streptavidin and preblocked (Sigma). Binding of biotinylated peptides was allowed to proceed at 20–22 °C for two hours with gentle shaking. The wells were washed three times with 300 µL of PBS-0.05% Tween. Each well was then loaded with 1 µg of recombinant NOS prebound with varying molar concentrations of NOS1 antibody for 1 h mixing at 4 °C in 100 µL of 2% BSA/PBS-Tween. The protein was incubated for 1 h at 20–22 °C with shaking, and the plate was washed three times, as before. Primary antibody at 1:3000 recognizing the *N*-terminus of NOS (Sigma) diluted in 2% BSA/PBS-t was added and incubated for 1 h at 20–22 °C with shaking, followed by washes. Secondary antibody at 1:25,000 in 2% BSA/PBS-t was incubated in the wells for 1 h at 20–22 °C with shaking, followed by three washes. The plates were loaded with enzyme chemiluminescent reagent, and positive results were detected on film. The film was scanned on a Kodak IS1000, and net intensity was determined. Alternatively, the plate was imaged directly on the Kodak IS1000, and image intensities were determined directly.

Visualization of protein–protein interactions—All protein interactions were evaluated using the crystal structures of 1v54.pdb and 1v55.pdb, analyzed by using RasMol and PyMol. Sequences that interacted with each subunit were found using Pfam, and the results were confirmed in the crystal structure. Alignments were performed with CLUSTALW. The search for PDZ domains was performed with SCANPROSITE. Search for class I or class II PDZ motifs, followed by turn-preferring residues, were performed with a custom-made string for SCANPROSITE based on turn preferences by ProtScale Tool (amino acid scale chosen as the normalized frequency for beta-turn [37] or as conformational parameter for beta-turn [38]).

Nitric oxide production—NOS activity was determined by following a modified version of a scintillation proximity assay [39]. This assay monitors the conversion of [^3^H]-L-arginine to [^3^H]-L-citrulline in 50 mM HEPES buffer (pH 7.4) in the presence of 1 mM CaCl_2_, 20 μg/mL calmodulin, 1 mM NADPH, 12 μM tetrahydro-L-biopterin, 170 μM dithiothreitol, 2 μM L-arginine, and 0.1 μM [^3^H]-L-arginine with or without the preincubation of NOS with the indicated peptide. The reaction was initiated by adding 10 nM of recombinant rat NOS1 enzyme (Calbiochem) and continued for 90 min at 20–22 °C. The reaction was stopped by adding 1 mg of scintillation proximity assay (SPA) arginine-binding beads (Amersham Biosciences) in 50 mM NaOH. Beads were allowed to settle for 2 h, and then the residual amount of [^3^H]-L-arginine in the reaction was determined by yttrium silicate SPA using a 1450 MicroBeta Liquid scintillation microplate counter.

Statistical analyses—Experiments were performed at least in triplicates (except ELISA experiments which were run in duplicates). The study shows representative images of technical replicates performed with at least three biological replicates.

## 3. Results

### 3.1. Identification of CCO Subunit IV as an Interacting Protein of NOS

To identify mitochondrial protein(s) that bind to brain NOS, we performed a standard immunoprecipitation procedure by incubating crude NOS (prepared from rat brain mitochondria) with mouse monoclonal antibodies to NOS type 1 (or NOS1; clone B1-Sigma, St. Louis, MO, USA) specific to the *N*-terminus of NOS. The antibody was prebound to protein-G agarose beads and blocked with BSA. A mock immunoprecipitation was performed by substituting the antibodies to NOS1 with purified preimmune mouse IgG. To confirm the specific immunoprecipitation of NOS, eluted fractions from agarose beads were assessed by Western blots by using specific antibodies to the *C*-terminal of NOS1 (R-20, Santa Cruz Biotechnology, Santa Cruz, CA, USA; Figure 1A). While at 5 µg and 10 µg of crude NOS1, the antibody directed to NOS1 showed the most discerning effect, at high loading, the specificity of the binding was lost, as expected (Figure 1A).

Once the specificity of the antibodies was confirmed by the above experiments, NOS1 from whole brain mitochondria lysates was immunoprecipitated with the R-20 NOS1 antibody. The precipitated complexes were separated by two-dimensional gel electrophoresis (second dimension at 15%), stained with SyproRuby™, and the digital images were analyzed by Delta2D version 3.2 software (AlphaTech, Geifswald, Germany). Spots present in the gel obtained with antibodies to NOS1 and not present in the mock gel were cut, trypsinized, and analyzed by MALDI-TOF MS. Utilizing the MW, p*I*, and the masses of the tryptic digests, the identification of the most abundant protein that immunoprecipitated with antibodies to NOS was performed. The tryptic peptides matched those of cytochrome oxidase subunit IV (SwissProt P10888; CCOIV). The peptides of this subunit, whose masses were obtained by MALDI-TOF MS, were underlined in the primary sequence, whereas the mitochondrial targeting sequence was highlighted (Figure 1B). The sequence coverage was 40.8%, supporting the validity of the procedure.

Two other proteins (with fewer fragments than those from CCOIV and likely less abundant) were also observed using this procedure, only obtained when the second dimension was run with 12.5% gel. This protein was identified as cytochrome *c* oxidase subunit V with peptides from both CCOVa and CCOVb. The finding of CCOVa confirmed a previous study in which NOS was found to interact with this subunit [23]. Still, it extended these protein–protein interactions to mainly CCOIV and other CCO subunits (Vb) not observed in the previous report. When immunoprecipitation was performed with cytosolic fractions instead of mitochondrial ones, phosphofructokinase-1 interacted with brain mitochondrial NOS, consistent with a previous report obtained with NOS1 [40].

Taken together, these results indicated the following possibilities: (i) that the immunoprecipitation of NOS–CCOIV may have pulled subunits V along (owed to the close packing of subunits within the cytochrome oxidase structure), without necessarily having a direct interaction with NOS; (ii) the reverse of the previous scenario (i.e., direct interaction of NOS with CCOV with coprecipitation of CCOIV); or (iii) that all three CCO subunits, i.e., CCOIV, CCOVa, and CCOVb interact independently and directly with NOS. In addition, these experiments, as with those by Persichini et al. [23], did not provide information on the putative requirement of calcium ions for the NOS–CCO interaction, given that the activation of NOS, as that of all constitutive NOSs, requires calcium ions and calmodulin.

### 3.2. Calcium Ions Are Required for NOS–CCOIV Protein–Protein Interaction

To discern among the possibilities indicated above, brain NOS bound to mitochondria was immunoprecipitated as indicated before, but in either the presence of 4–6 nmol calcium ions/mg mitochondrial protein or 4 mM EGTA. These calcium ions concentrations were chosen because they fully activate NOS localized in mitochondria [35,41,42], and are within the range of the increases in calcium ions in the mitochondrial matrix when exposed to various stimuli (see references [35,41] for specific examples). Calcium ions were found to be required for the interaction between CCOIV and NOS. This was supported by the detection of CCOIV in Western blots when the immunoprecipitations were performed in the presence of calcium ions and absent when EGTA was present (Figure 1C, top panel).

Western blots were also performed to determine if the immunoprecipitation of NOS with CCOIV entailed interacting with any of the catalytic subunits (CCOI, CCOII, and CCOIII). Western blots using antibodies to CCOI indicated that this subunit did not coimmunoprecipitate with NOS regardless of the presence of calcium ions or EGTA (Figure 1C, bottom panel). Since commercially available antibodies to human CCOII and CCOIII did not crossreact with the corresponding rat subunits, custom-made antibodies to rat CCOII and CCOIII (ProSci; San Diego, CA, USA) were prepared. The former one crossreacted with both rat and mouse CCOII; but as the MW of the mature CCOII is almost the same one as that of the antibody light chains (about 26 kD), it was not possible to obtain a conclusive answer for this subunit. Despite many attempts, the CCOIII antibody showed weak binding to rat CCOIII, thus preventing further analysis.

To explore the putative need for calcium ions for the NOS–CCOIV interaction, recombinant NOS1 was biotinylated and bound to immobilized, avidin-linked beads. The agarose column was blocked with biotin, and subsequently incubated with brain mitochondrial inner membrane lysate supplemented with either 4–6 nmol calcium ions/mg mitochondrial protein or 5 mM EGTA and washed extensively with 1 M NaCl in acetate buffer until no proteins were detected on silver-stained SDS-PAGE (Figure 1E). The degree of purification of the mitochondrial inner membrane was obtained by evaluating the mitochondrial fractions and isolated submitochondrial fractions of several specific enzymes. The recovery of markers of different compartments for the inner membrane preparation was (of purified mitochondria) 0.002% Cu,Zn-superoxide dismutase (as a marker of intermembrane space and cytosol); 0.06% monoamine oxidase (outer membrane); 1.9% Mn-superoxide dismutase (matrix); 0.01% adenylate kinase (intermembrane space); and 43.5% cytochrome *c* oxidase (inner membrane). Further analysis confirmed the purity by using Western blots directed to markers of mitochondrial subcompartments (Supplementary Figure S1). Therefore, the inner membrane preparations utilized in this study were of high quality and purity. Proteins tightly bound to the column were eluted sequentially with elution buffer supplemented with EGTA. The eluted proteins were subsequently analyzed by Western blotting (Figure 1E). Our results indicated that the binding of CCO to NOS required the presence of calcium ions, whereas the release of this interaction was promoted in the absence of this cation. This conclusion was supported by the presence of CCOIV in the eluted fraction only if the binding to the column was performed in the presence of calcium ions and eluted with EGTA (Figure 1E, top two panels). In all other cases, CCOIV was not detected because either it was not released from the column (if calcium ions were present throughout the procedure) or eluted with the washes (when EGTA was present either during binding or during binding and elution; Figure 1E, bottom two panels). When these results were compared to control experiments in which a column (with no biotinylated NOS) was incubated with inner mitochondrial membrane lysates to identify nonspecific binding proteins (even though several proteins were nonspecifically bound to the column and eluted from it), Western blots performed on these eluates showed no CCOIV, indicating that the interaction between NOS and CCOIV was specific and calcium-dependent (Figure 1E, all panels under “Control”). To confirm the NOS–CCOIV interaction, CCOIV was immunoprecipitated from a crude preparation of NOS using a mouse monoclonal antibody to CCOIV (Molecular Probes, Eugene, OR). This immunoprecipitation also pulled down NOS, whereas preimmune IgG did not (Figure 1F). These data indicated that CCOIV and NOS interacted and could be coimmunoprecipitated, suggesting that a significant fraction of NOS in rat brain mitochondria is associated with CCOIV.

Instead of serving as a requirement for binding NOS to CCOIV, calcium ions may help recruit NOS to the mitochondrial membranes. If this were the case, the recruitment of NOS would facilitate the interaction with CCOIV and strengthen the need for having a source of NO^•^ close to the target site, CCO. To this end, experiments were performed by incubating mitochondria lysates in the presence of either 4–6 nmol calcium ions/mg protein or 20 mM EGTA, and the recruitment of NOS to mitochondrial membranes was recorded by performing Western blots of 150,000× *g* pellets (thoroughly washed with KCl and CHAPS, supplemented with either calcium ions or EGTA if the original incubations contained one or the other) with antibodies to NOS and antibodies to beta-subunit of ATPase (as loading control; Supplementary Figure S2). The OD ratios expressed as OD NOS/OD beta-subunit under each condition were not significantly different (mean ± SD for calcium 1.1 ± 0.2 and EGTA 1.2 ± 0.3, *n* = 6; Student’s t-test paired samples followed by Tukey’s *p* < 0.09). These experiments demonstrated that calcium ions are required for NOS’s activation and binding to CCOIV but not for its recruitment to the membranes.

Furthermore, no statistically significant changes were detected in the respiratory control ratio (RCR; i.e., the ratio of oxygen uptake of phosphorylating and nonphosphorylating mitochondria) of the mitochondrial preparations (3.9 ± 0.5 vs. 3.0 ± 0.1) at these calcium concentrations needed to exhibit full activity [35,41,42]. Given that ADP does not freely cross the mitochondrial inner membrane, we can safely assume that the mitochondria were not uncoupled by calcium ions or that calcium ions at the concentrations did not promote the formation of significant pores (consistent with other studies [43,44,45,46]). Uncoupling (or changes in the RCR) is extremely sensitive to changes in mitochondrial membrane intactness, even when small pores are induced. For instance, alamethicin treatment completely uncouples mitochondria by promoting the formation of 10 to 20 Å pores and allowing the passage of molecules < 3 kD [47,48]. Thus, the lack of changes in the RCR of mitochondria with and without these calcium ion concentrations strongly suggests functional integrity and eliminates the possibility of a calcium-dependent increased mitochondrial membrane permeability.

### 3.3. NOS Binds Directly to the Matrix Side of CCOIV

PDZ domains were originally recognized as ~90 amino acid long repeated sequences in the synaptic protein PSD-95, the *Drosophila* septate junction protein Discs-Large (Dlg), and the epithelial tight junction protein ZO-1 [49]. They are among the most common protein domains, and in addition, PDZ domains show considerable sequence variation, presumably reflecting their diversity of binding specificities and functional roles. Several discoveries defined the primary function of PDZ domains as protein interaction domains that bind in a sequence-specific manner to short COOH-terminal peptide motifs. Although *C*-terminal peptides are the typical ligands (class I = X-S/T-X-V/L, class II = X-f-X-f, and class III = X-D-X-V, where f is a hydrophobic residue), PDZ domains can bind to internal peptide sequences that bend in a beta-hairpin structure [50] and to other PDZ domains. Given that CCOIV contains in its primary sequence neither a *C*-terminal peptide that could act as a ligand for the NOS-PDZ domain nor a domain that docks the beta-hairpin structure of NOS1 [50], we investigated the interaction of NOS with CCOIV by following different approaches.

**Figure 2:**
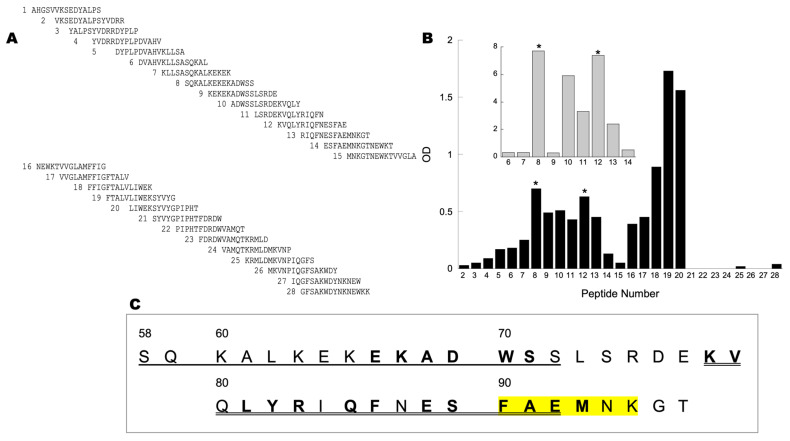
Brain NOS binds to specific peptides within CCOIV. Panel (**A**) shows the library of overlapping biotinylated peptides spanning the mature CCOIV utilized to scan the interaction of NOS with CCOIV. Each of the 28 peptides (each 15 amino acids long and biotinylated at the *N*-terminus). Panel (**B**) shows far Western blots of NOS with CCOIV peptide library. Proteins in the crude (fresh) NOS preparation (10 µg) were separated on denaturing gels, and transferred to PVDF membranes. Protein lanes were cut into separate strips, and each was incubated with one peptide (1.5 μg/mL for 1 h at 20–22 °C with an orbital shaker). The strips were incubated with HRP-conjugated avidin. The bands were visualized using Pierce Supersignal West Femto on a Kodak IS 1000 imager. The net intensity of each NOS band was normalized to that of avidin-binding pyruvate carboxylase. Inset: Far Western protocol for recombinant NOS: Recombinant NOS1 (1 µg, rNOS1) per lane was separated and transferred as described above. The strips were incubated with peptides 6–14, then visualized and processed as described above. Negative controls consisted of rNOS1 without peptides with avidin-HRP. Asterisks indicate significant peptide binding. Panel (**C**) shows the amino acid sequence of CCOIV from peptides 8 through 13. Underlined and double-underlined segments indicate peptides 8 and 12, respectively. Residues in bold interact directly with CCOVa, as determined by X-ray structure analysis and Pfam interactions. Highlighted residues interact with the *C*-terminus end (ELGLDKV) of CCOVa. The *C*-terminus of CCOVa contains a PDZ ligand class III consistent with the following sequence XDXV.

To evaluate the binding site of NOS on CCOIV, we designed 28 peptides that spanned the complete primary sequence of mature CCOIV. Each peptide was 15 amino acids long, staggered by 5 contiguous residues, labeled at the *N*-terminus with biotin (Figure 2A). Recombinant NOS1 (Figure 2B inset) or crude brain mitochondrial NOS (Figure 2B main panel) was incubated with each of the 28 peptides in solution. Each mixture was developed by slot-blots using streptavidin-HRP conjugates. Software analysis of the slot-blots digital images indicated that the strongest interaction of recombinant NOS1 was obtained with peptides 8 and 12 (residues 56–70). Similar results were obtained with the crude brain NOS, which interacted significantly with peptides 8 and 10 through 12 (residues 46–70; Figure 2B). The transmembrane region of CCOIV (Figure 2C, peptides 16–20) was expected to interact with NOS through unspecific hydrophobic contacts.

Since the background of slot-blots performed with crude brain NOS was higher than those using recombinant NOS1 due to the presence of endogenous biotin-containing proteins (Supplementary Figure S3), we tested the binding of the peptides to recombinant NOS1 or brain mitochondrial NOS by using a far Western protocol. Crude brain mitochondrial NOS was separated by 7.5% SDS-PAGE and transferred to PVDF. The blot was then cut into strips, and each strip was incubated individually with each of the peptides from the set. The binding of the biotinylated peptide could then be specifically analyzed and compared to the biotin-containing pyruvate carboxylase as an internal loading control (Figure 2B). Under these conditions, recombinant NOS1 and brain NOS from mitochondria interacted significantly with peptides 8 and 12 (Figure 2B with asterisks), in agreement with the binding pattern obtained with slot-blots.

When the amino acid sequence for the recombinant NOS1 binding peptides is depicted (Figure 2C), it becomes clear that there are two options for the NOS–CCOIV interaction. One option involves a displacement of CCOVa from CCOIV to accommodate NOS, for several residues within these CCOIV segments are involved in the direct binding with CCOVa (Figure 2C, bold residues). The other option includes a binding site for NOS independent of CCOVa. Mammalians have two isoforms of CCOIV, IV-1 and IV-2, with an overall primary sequence homology of about 44% (humans, rats, mice). Interestingly, those residues implicated in the binding to NOS are 83% conserved across species (Figure 2C), suggesting that the binding of NOS can occur with any of the CCOIV isoforms. However, while liver and lung do express both isoforms, in the brain, only one is present (COXIV-1, COXIVa, or COX4I1) [51]. This isoform lacks the 8 bp proximal promoter HIF-1 binding motif, so in a highly aerobic tissue such as CNS, it makes sense to have a CCO with full activity that is not sensitive to oxygen levels (as is the case for lung and liver, for instance; see normalized expression levels of COX4I1 and COX4I2 for 55 tissue types created with The Human Protein Atlas [52] under Supplementary Figure S4).

Within the interacting residues, a highly conserved sequence (ALKEK) located in peptide 8 has no contact with any other subunit. It is directly accessible to the matrix, making it one of the likely motifs for the NOS–CCOIV interaction.

**Figure 3:**
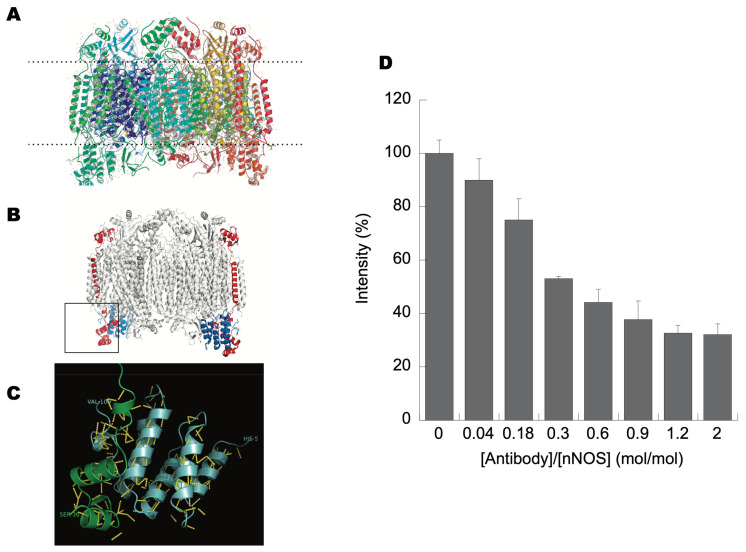
Three-dimensional structure of cytochrome oxidase. In Panel (**A**), the 3D structure was performed by visualizing the pdb file with RasMol, later refined using ChemDraw 3D Ultra and PyMol. Panel (**A**) shows the structure of cytochrome oxidase with all 13 peptides (based on 1v54.pdb); Panel (**B**) shows subunits IV and Va indicated as red and blue, respectively; Panel (**C**) shows the C-terminus of CCOVa (light blue) and the segment of CCOIV covered by peptides 9–13 (green). In yellow, polar interactions between chains. Panel (**D**) shows competitive displacement of peptide 12 (from CCOIV) bound to NOS1 with *N*-terminus antibody to NOS. Streptavidin-coated plates were prebound with peptide 12. Recombinant NOS (1 µg) was incubated with increasing *N*-terminal NOS antibody molar ratio from (0.04 to 2.0). ELISA plates were washed with PBS-t after peptide binding and then incubated with prebound NOS1-antibody complexes (1 µg of prebound NOS1 per well). Plates were extensively washed and incubated with *N*-terminal NOS1 antibodies to visualize NOS1 attached to peptide 12. Plates were again washed and incubated with ECL reagents to determine the net intensities of each well. Wells bound with peptide 12 (shown) or 8 (not shown) and incubated with 1 µg of recombinant NOS1 not prebound with antibody were considered 100%.

The three-dimensional structure of CCO (Figure 3A, top panel) and localizing CCOIV in this 3D structure of bovine heart cytochrome *c* oxidase, as determined by electron crystallography to 20-Å resolution, reveals that CCOIV has three domains: an *N*-terminus matrix domain, an alpha helix that constitutes the transmembrane domain, and a *C*-terminus domain that protrudes into the intermembrane space (Figure 3B, middle panel). By selecting the sequence that interacted with NOS in the 3D structure of CCOIV (Figure 3B, middle panel, square), a motif is highlighted towards the *C*-terminus end of CCOIV. This motif includes three alpha helices intercalated by two random coils, ending closely to the inner leaf of the inner membrane. Given the localization of this binding domain, NOS likely interacts with CCOIV on the matrix side. These results are consistent with earlier studies that showed a colocalization of NOS with CCO in liver slices [13] and that NOS in mitochondria was preferentially found at the inner membrane and contact sites [12], indicating that these proteins shared the same subcellular compartmentalization, a requirement for a suitable protein–protein interaction. It is also clear from X-ray structures, as well as from Pfam studies, that the *C*-terminus of CCOVa is not free but interacts directly with CCOIV (Figure 3C), preventing the direct binding of any protein to the CCOVa *C*-end, as initially proposed by Persichini et al. [23]. While the CCOIV–NOS interaction may cause CCOVa to expose its *C*-end, allowing NOS to interact with CCOVa via its PDZ domain, the immunoprecipitation of CCOVb always carried both proteins CCOIV and NOS. Thus, the most likely explanation is that the presence of CCOV in the second dimension run at 12.5% reflected the interaction of CCOIV with CCOV, since subunit CCOVb interacts with CCOIV at segments not present and away from peptides 8–12.

### 3.4. N-Terminus End of NOS Binds CCOIV

NOS1 is targeted to the synaptic membranes in the brain through interactions of its postsynaptic density-96 (PSD-95) and PSD-93 [38,39]. PSD-95 and PSD-93 are major proteins of the postsynaptic density and bind to NOS1 via PDZ (PSD-95, discs-large, ZO-1) domains, the consensus sequence of approximately 100 amino acids found in some cytoskeletal proteins and enzymes that mediate protein–protein interactions [53]. The association of NOS1 and PSD-95 is mediated by a direct PDZ–PDZ interaction [54].

Given that NOS localized in rat brains is the alpha NOS1 isoform, we investigated whether this interaction required the PDZ domain of NOS. Considering our previous results—from slot-blot, immunoaffinity chromatography, and far Western blots—it is concluded that the interaction between NOS and CCOIV does not include a PDZ–PDZ interaction, for CCOIV does not have this domain in its primary sequence. To determine experimentally whether CCOIV binds to the *N*-terminus of NOS1, we used an ELISA competition assay to assess the binding of recombinant NOS1 to CCOIV with antibodies directed towards the *N*-terminus of NOS1 (residues 1–181). First, the biotinylated peptide 12 was bound to 96-well plates coated with streptavidin. Recombinant NOS1 was preincubated with antibody to the *N*-terminal epitope of NOS1 at increasing molar ratios of antibody to protein and then allowed to bind peptide 12 of CCOIV on the plate. The binding to peptide 12 was abolished by 70% with antibodies directed towards the *N*-terminus of NOS (residues 1–181) when used at a molar ratio of 1.2:1 antibody to NOS (Figure 3D). These results suggested that the binding of CCOIV is at the *N*-terminus of NOS. This binding domain lies outside the NOS1 regions that bind calmodulin, cofactors, and substrates. To reconcile the observation of Persichini et al. that CCOVa binds to NOS at its PDZ domain (residues 17–99 [23]) with the results presented here, the binding of NOS to CCOIV would have to be via either the PIN binding site (10 kD protein inhibitor of neuronal nitric-oxide synthase [55]; DYNLL1) at residues 163–240 or in between the PDZ and PIN binding sites (residues 100–162).

### 3.5. NO^•^ Production Is Decreased by CCOIV Interaction but Still Decreases ATP-Linked Oxygen Uptake by Isolated Brain Mitochondria

**Figure 4:**
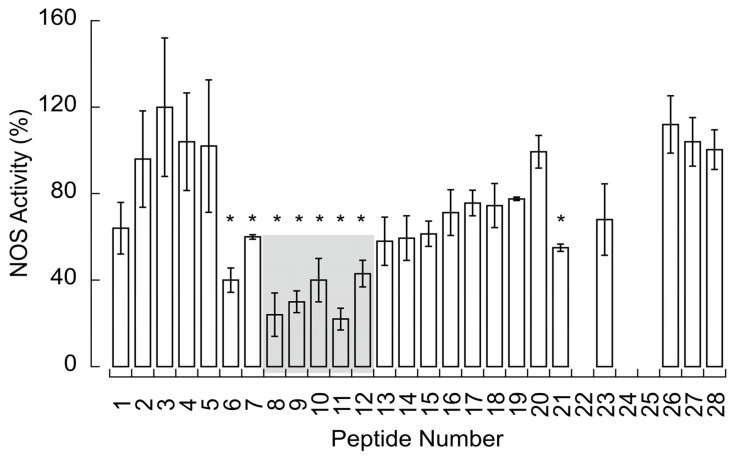
Effect of CCOIV peptides on NOS activity. Recombinant NOS1 was incubated with equimolar amounts of overlapping biotinylated peptides from CCOIV library (shown in Figure 2A) in 50 mM HEPES buffer (pH 7.5) containing 0.2 mM dithiothreitol for 1 h at 20–22 °C. The SPA assay was used to evaluate NOS activity (described in detail under Methods). Experiments were run in triplicates and on two different occasions. Values are presented as mean ± SEM, and 100% represents the NOS activity without any peptide under the exact experimental conditions. Statistical analyses were performed using one-way ANOVA followed by Fisher’s least significant comparison test (* *p* ≤ 0.05 vs. peptides 2–5). Shown in grey are peptides involved in the CCOIV–NOS binding (from Figure 2B). The lack of NOS activity observed with some peptides was due to the inhibitory effect of DMSO used as a vehicle for the most hydrophobic peptides.

To elucidate the functional implication of the NOS–CCO interaction depicted above, the NO^•^ production by recombinant NOS1 was tested in the presence of the different COXIV peptides (Figure 4). This production was (mean ± SEM) 32 ± 4% of the full activity upon binding those peptides involved in the CCOIV–NOS interaction (Figure 4 in grey).

To test whether this production modulated the ATP-linked oxygen production, isolated brain mitochondria were incubated with N^G^-monomethyl-L-Arg (NMMA; competitive inhibitor of NOS) or L-Arg (to promote NO^•^ production by NOS). The oxygen uptake rate in State 3 in the presence of alpha-ketoglutarate as a substrate was 58 ± 4 nmol oxygen × (min × mg protein)^−1^ with NMMA. In the presence of L-Arg, the rate was decreased by ~40% (35 ± 3 nmol oxygen × (min × mg protein)^−1^). Reactions mixtures supplemented with calcium ions (6–8 nmol/mg) were increased (75 ± 3 nmol oxygen × (min × mg protein)^−1^) or decreased (28 ± 3 nmol oxygen × (min × mg protein)^−1^) with NMMA and L-Arg additions, respectively (Supplementary Figure S5). With no addition of calcium or L-Arg, the NO^•^ production was 2.0 ± 0.5 nmol × (min × mg protein)^−1^, whereas that with L-Arg and calcium ion addition was 5.5 ± 0.5 nmol × (min × mg protein)^−1^. No significant rates were obtained with NMMA with or without calcium. The 1.3-fold increase in State 3-linked respiration in the presence of NMMA with calcium supplementation vs. the one without it can be interpreted as the effect of calcium on the Krebs’ cycle dehydrogenases’ activation. The increase is within that reported by us [35] and others [56]. Adding L-Arg promotes NO^•^ production (with endogenous or exogenous calcium ions addition), decreasing the oxygen uptake rates by 40% and 52%, respectively.

## 4. Discussion

The primary finding of this study is that the matrix side of the subunit CCOIV from Complex IV binds to the *N*-terminus of NOS in a calcium-dependent manner and modulates the activity of CCO via NO^•^ production. The location of CCOIV, the largest nuclear-encoded subunit of Complex IV, and its contact sites with catalytic subunits I and II make it a likely candidate for modulating the terminal oxidase activity. The NOS–CCOIV interaction may lead to CCO activity modulation under various *p*O_2_. Indeed, we showed that CCOIV peptides implicated in the NOS–CCOIV binding decreased the NOS activity by 70%; however, even at this decreased rate of NO^•^ production, NO^•^ still modulates CCO activity (as shown in isolated mitochondria). This modulation may have two implications: one, by decreasing CCO activity under hypoxic conditions (instead of a complete halt), it ensures that still electrons are flowing through the respiratory chain, minimizing the formation of partially reduced oxygen species [18], and two, it allows oxygen to diffuse away to other proteins with higher *K*_m_ for oxygen [22].

Other studies reported that nuclear-encoded subunits of CCO (including CCOIV) and their isoforms regulate CCO activity in an NO^•^-independent manner and without being directly involved in reducing oxygen to water [57,58]. For instance, the allosteric ATP modulation of CCOIV is crucial in higher organisms [59,60]. CCOIV binds ATP at the matrix side, leading to allosteric inhibition of CCO at high matrix ATP/ADP ratios [61], an effect further modulated through cAMP-dependent phosphorylation of subunits II and/or III and Vb [62]. In contrast to higher eukaryotes, CCO from *S. cervisiae* contains six nuclear-encoded subunits; among them, subunits Va and Vb (encoded by *COX5* genes), with a primary sequence homologous to the mammalian CCOIV [51]. Changes in CCO activity, coupled with the oxygen-dependent regulation of the Vb-to-Va ratio, would allow considerable control of oxidative metabolism over a wide range of oxygen concentrations as the *COX5* genes are regulated differently by oxygen and heme [63,64,65,66,67].

A striking result of our work is that the binding of CCO to NOS requires calcium ions. The NOS–CCO interaction occurs at calcium ion concentrations higher than those required to activate Krebs’ cycle dehydrogenases (6 to 7 vs. 1.5 to 2 nmol/mg mitochondrial protein, respectively). This activation decreases the oxygen uptake via NO^•^ production. It could be argued that calcium ions may inhibit CCO activity directly [68]; however, the mol-to-mol ratio of calcium ions to CCO to achieve this effect is >3000 (calculated from [68]), whereas that for the NOS–CCO interaction is in the order of 500 calcium ions/*aa*_3_ heme.

At this point, we do not know the exact mechanism by which calcium ions affect the binding, but several scenarios can be presented: one, calcium ions may minimize charge repulsion at residues in the NOS–CCOIV contact site; two, calcium ions may fix protein segments to secure an effective docking; or three, calcium ions, by binding to the intermembrane segment of CCOIV (i.e., transmembrane domain), may induce a conformational change, transmitted along its chain, allowing the binding of NOS to CCOIV. After this interaction, CCOIV changes in conformation could elicit the exposure of the CCOVa *C*-terminal peptide, allowing the NOS PDZ domain to interact with it. This PDZ domain is present in four of the five isoforms of NOS1 produced by alternative splicing (Supplementary Figure S6) and not in NOS2 or NOS3. Thus, the interaction of NOS with CCOIV can occur with all isoforms except isoform 3 (P29475-3). Still, only those having the PDZ domain will abide by the CCOV interaction, consistent with the proposed mechanism by Persichini et al. [23]. The NOS–CCO interaction might directly regulate cellular bioenergetics. Calcium ions influx, by activating NOS, may facilitate the docking of NOS to CCO, thus positioning the production of NO^•^ close to the target site, requiring less NO^•^ to be generated. Doing so minimizes deleterious side reactions and maximizes the effect of NO^•^ on the oxidase or other putative mitochondrial targets. When calcium ion levels are restored to basal conditions, the deactivation of NOS is achieved, and the release of the enzyme from CCOIV. Thus, this provides a dynamic mechanism for controlling the oxygen consumption rate upon calcium ion concentration changes via the activation and docking of NOS to CCO.

NO^•^-related effects on mitochondrial respiration and function and mitochondrial NO^•^ production has been reported in various biological systems [10,13,14,22,42,69,70,71,72,73,74,75,76,77,78,79,80,81,82,83,84,85,86,87,88] utilizing a variety of techniques (immunohistochemistry, immunofluorescence, electron microscopy, confocal microscopy, electron paramagnetic resonance, colorimetric or fluorescent probes, and electrochemistry [13,71,72,89,90,91,92]), including KO mice [93]. While these investigations have provided valuable insights, it is essential to acknowledge that, like all scientific studies, they come with their own limitations and advantages [94,95,96,97]. Adding to these points, no NOS is reported in MitoCarta4.0 (1136 human proteins [98]), the most comprehensive, validated inventory of mitochondrial proteins. However, an additional (not reported in MitoCarta yet) ~400 putative proteins are listed under an integrated mitochondrial protein index under Mitominer V4.0 Field [36], with most awaiting experimentally validation. The UniProtKB database has 2880 reviewed human mitochondrial proteins, with 16,630 still needing to be checked or verified [99]. Then, it could be argued that many mitochondrial proteins (also known as mitochondrial orphans) still need to be reported [100]. Advanced technologies are allowing a more nuanced and precise proteomic profile of mitochondrial subcompartments [101,102,103]; however, we still need to consider that a plethora of factors (including metabolic states, medical conditions, sex, age, tissue of origin, and subcellular organelle distribution) also play a role in shaping the mitochondrial proteome of these dynamic organelles. In sum, while cumulative evidence suggests NO^•^ production within mitochondria and its potential role in mitochondrial function, a distinct and dedicated NOS isoform remains a subject of ongoing investigation. In this regard, improved and specific imaging technologies have reported specific NO^•^ production by mitochondria in several cell types and organisms [104,105,106].

Regarding the broader implications of this study, we need to consider the case of neuronal mitochondria and brain calcium ions homeostasis. For instance, the distribution of neuronal mitochondria is responsive to immediate and local physiological conditions, such as ATP synthesis needs and regulation of calcium homeostasis. The pyruvate formation is decreased at low cytosolic calcium ions (<100 nM), providing a substrate limitation control in neurons [107]. In the 100–300 nM cytosolic range, calcium ions enhance oxidative phosphorylation once they are taken up by the mitochondrial calcium uniporter (MCU) or the AGC1/Aralar component of the aspartate–glutamate carrier, activating several Krebs cycle dehydrogenases [108,109,110]. Other studies expanded this effect by reporting a calcium-dependent increase in the conductance of Complexes I, III, IV, and V [111]. Calcium ions activate the State 3 respiration (phosphorylating mitochondria) in neurons via AGC1/Aralar [112] when glucose is available [107,113], even when cytosolic calcium ions levels are below the activation range of the mitochondrial calcium uniporter [114]. This mechanism promotes or accelerates glycolysis and favors the formation of pyruvate for its oxidation in mitochondria by activating several Krebs cycle dehydrogenases [108,109,110]. If glucose is replaced by lactate–pyruvate or pyruvate alone, then a major role of MCU in upregulating ATP production is observed [114], suggesting similar yet complementary mechanisms. At even higher cytosolic calcium ion levels (>500 nM), mitochondria take up calcium for storage, activate substrate oxidation, and produce ATP to sustain the activity of calcium-dependent ATPases. Mitochondria located close to these high-rise calcium [115,116], such as those near the opening of IP3Rs and RyRs [117], are exposed to concentrations ~10-fold of the total cytosolic calcium ion concentrations [118,119]. These high calcium levels from IP3R/RyR inhibit mitochondrial movements [120]. Then, to accommodate the plasticity of axonal and synaptic structure and function undergoing spontaneous and activity-dependent remodeling and changing the demand for mitochondria, motile mitochondria are recruited to the stationary pool near synapses in response to elevated cytosolic calcium ions and synaptic activity [120,121,122]. Identification of mitochondrial motor-adaptor transport complexes KIF5-Milton-Miro [123,124,125,126], KIF5-syntabulin [127], and SNPH-DYNLL1 anchoring to cytoskeleton [128], with Miro serving as a calcium sensor that controls mitochondrial movement [123,124,129], provide molecular targets for such regulation. These mechanisms enable neurons to maintain proper densities of stationary mitochondria within axons and near synapses (see review [130]).

Within this background of information on the management of calcium and neuronal mitochondria, we could hypothesize that at high-rise calcium spots, docked, stationary mitochondria ideally serve as local energy stations by providing ATP to sustain the high activity of Na^+^-K^+^ ATPases while maintaining calcium ion homeostasis to support synaptic transmission. But as the NO–CCO interaction is enhanced, this process is accompanied by maintaining NADPH-dependent antioxidant defenses to minimize side damaging reactions. This is accomplished by balancing the need for ATP (activation of dehydrogenases) while sustaining [NADH]/[NAD^+^] and [NADPH]/[NADP^+^] ratios (decreased electron flow through CCO). Additionally, the oxygen gradient is extended to other mitochondria, not necessarily located at the high-rise calcium spots, thus decreasing the steepness of the oxygen gradient formed between high-oxygen consumers (near calcium hotspots) and low-oxygen consumers (away from these sites [22]). Alternatively, it is possible that the reduced ATP production, modulated by NO^•^, drops below the level required to support ATP-dependent kinesin motors responsible for moving cellular cargo in the forward direction along microtubules. This situation could lead to promoting or aiding the retention of stationary mitochondria. As the coordination of mitochondrial mobility/docking with axonal physiology is crucial for neuronal and synaptic function, it is not surprising that defective transport and docking of axonal mitochondria is implicated in human neurological disorders and neurodegenerative diseases [131,132,133], particularly affecting neurons with long axonal processes (i.e., motor neurons).

Furthermore, perturbations in calcium homeostasis were reported in neurodegeneration and several neurodegenerative disorders, including Alzheimer’s disease, Parkinson’s disease, Huntington’s disease, and amyotrophic lateral sclerosis [134,135,136]. Calcium homeostasis disruption implicates several mechanisms, such as alterations of calcium buffering capacities, deregulation of calcium channel activities, or excitotoxicity. As dysfunctional mitochondria (from an existing disease, e.g., Parkinson’s) may exhibit increased free radical production and decreased ATP production, including increased intracellular calcium concentrations, then it would be expected that some of the cellular damage may result from NO^•^-CCO by, for instance, increasing side-damaging reactions that may exacerbate or propagate even further the neurodegenerative process.

## Data Availability

All data are included in this study.

## Supplementary Materials

The following supporting information can be downloaded at: https://www.mdpi.com/article/10.3390/brainsci13111534/s1, Supplementary File S1: CCOVa protein interactions; Supplementary Figures: file with six supplementary figures.
