# Supplementary material for: Calcium-Dependent Interaction of Nitric Oxide Synthase with Cytochrome c Oxidase: Implications for Brain Bioenergetics
Source: Brain Sci. 2023 Oct 31;13(11):1534. doi: 10.3390/brainsci13111534 (PMC10669843; doi:10.3390/brainsci13111534)

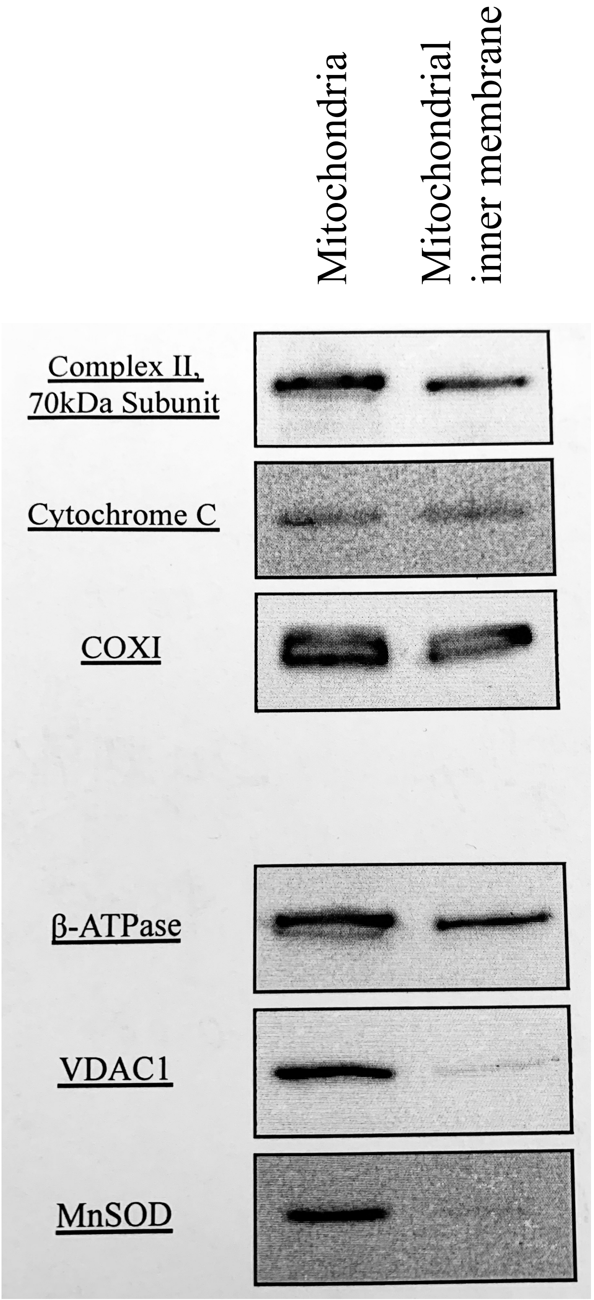

Supplementary Figure 1
A composite of representative Western blot images to test the purity of inner membrane fractions. Each lane contained 25 µg protein. After transferring the proteins onto a membrane, it was cut (based on the MWM) to probe for each antibody separately. The biomarkers tested by antibodies were Complex II 70-kD subunit, cytochrome *c*, CCO subunit I, Complex V subunit beta; VDAC1; and MnSOD. The Western blot method was described in the main text.

Supplementary Figure 2

Representative Western blot images (from a notebook) to test for the effect of calcium on the binding of a crude NOS preparation to membranes. Densitometry of NOS band normalized to that of ATPase beta subunit was used to perform the statistical analysis across several images.

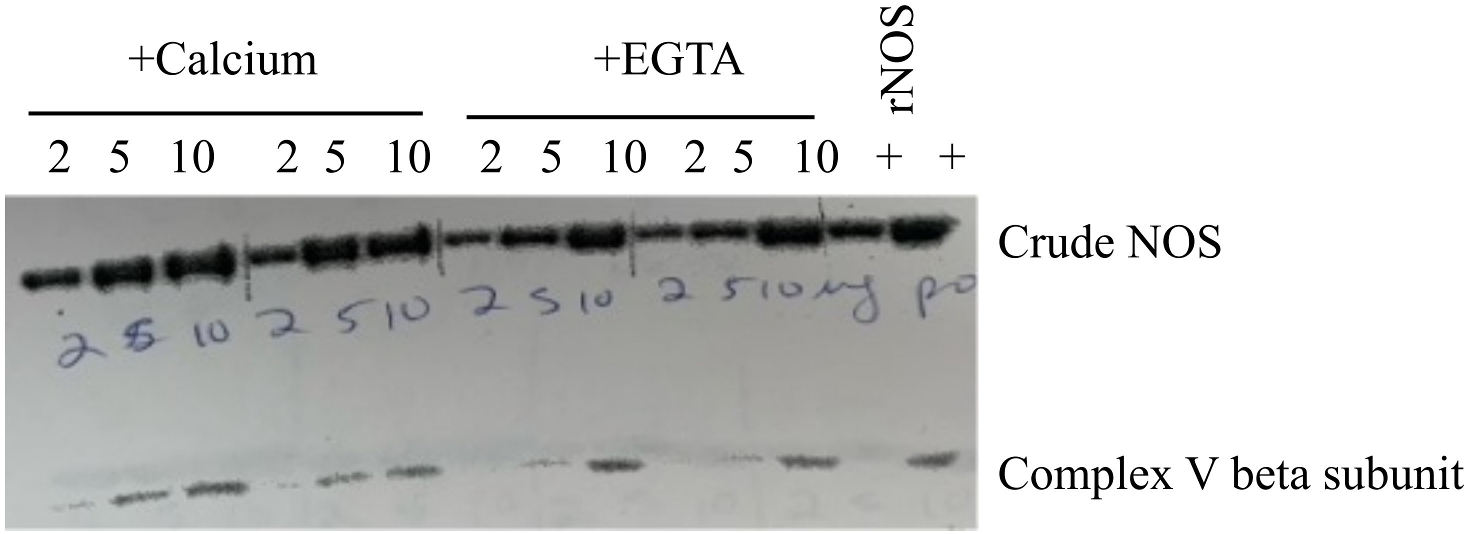

Supplementary Figure 3.

Representative Western blot images (from a notebook) from the strips incubated with crude brain NOS and the corresponding peptide (indicated with numbers). Densitometry of NOS band normalized to that of pyruvate carboxylase (PC) was used to perform the statistical analysis across several images collected from different experiments and strips.

6 7 8 9 10 11 12 13 14 15 16 17 18 19 20 21 22 23 24

Crude brain NOS

PC

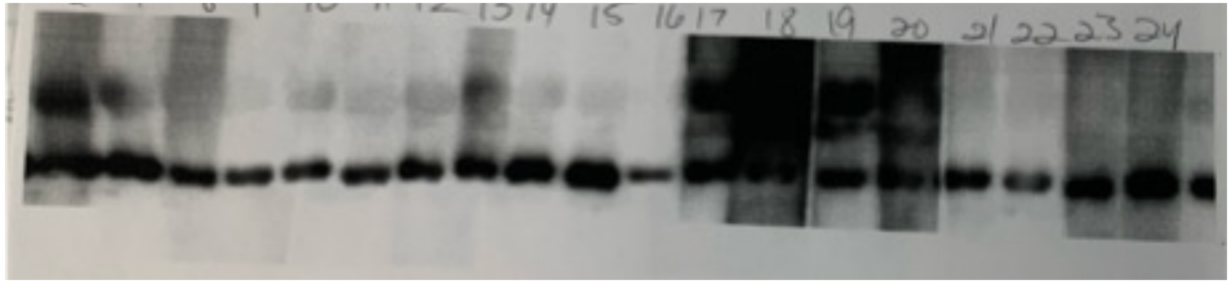

COX4I1

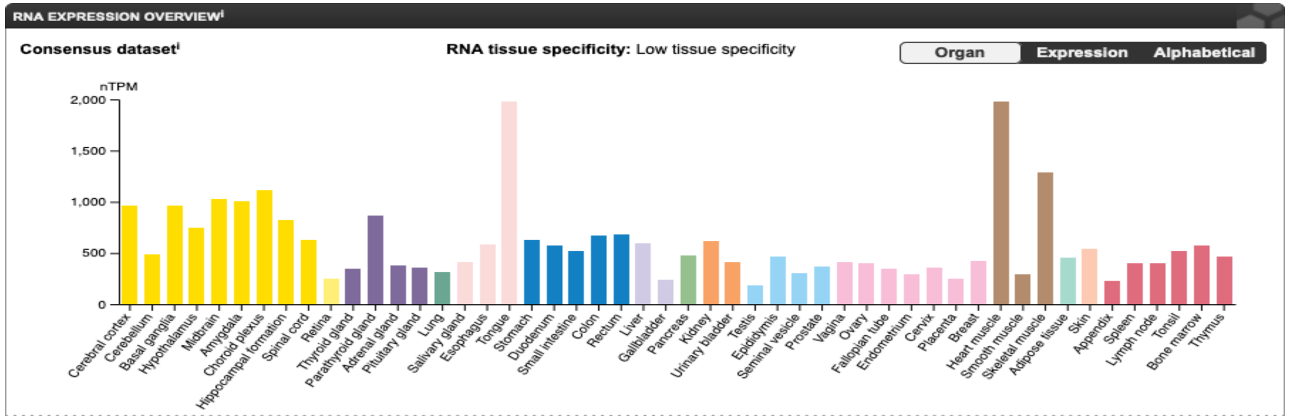

COX4I2

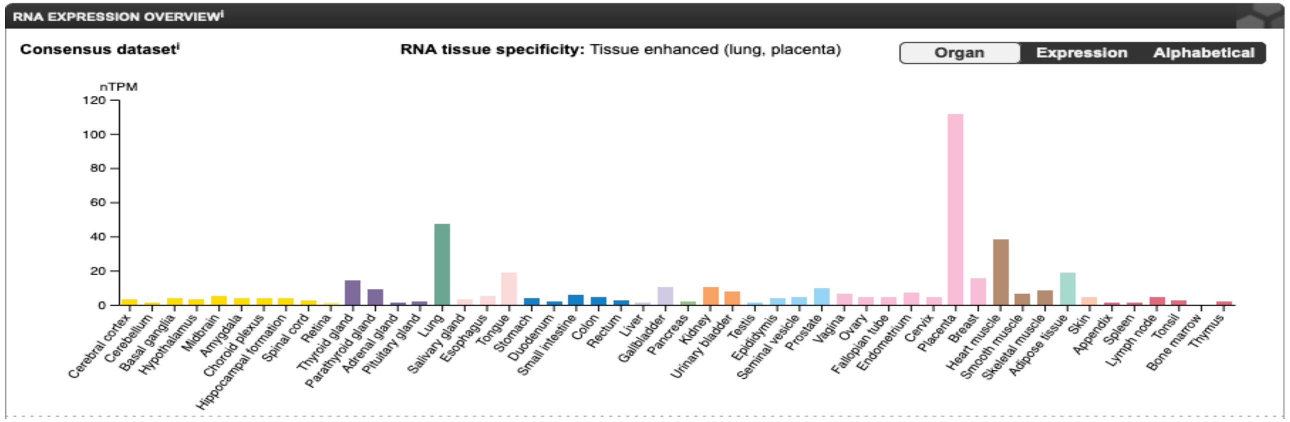

Normalized expression levels of COX4I1 and COX4I2 for 55 tissue types, created by combining HPA and GTEx transcriptomics datasets. Color coding is based on tissue groups, each consisting of tissues with functional features in common.

Reproduced from The Human Protein Atlas www.proteinatlas.org.

Images available at https://www.proteinatlas.org/ENSG00000131143-COX4I1/tissue#rna_expression and https://www.proteinatlas.org/ENSG00000131055-COX4I2/tissue#rna_expression).

Supplementary Figure 4

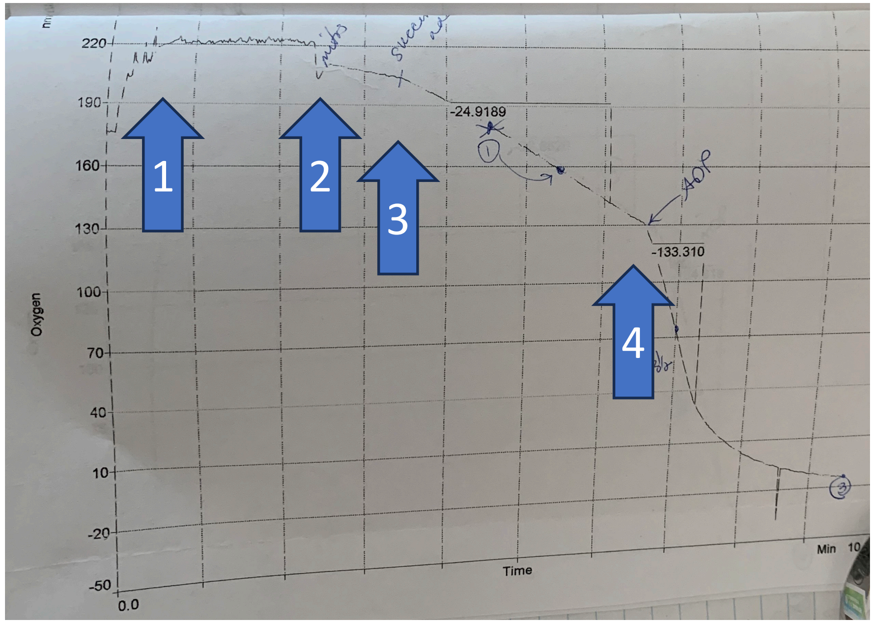

Supplementary Figure 5

A. Representative oxygen uptake trace obtained from one of the notebooks performed with purified brain mitochondria in reaction buffer. Indicated with numbers: (1) Buffer alone; (2) addition of 1.3 mg protein/ml of mitochondria; (3) addition of 10 mM succinate; (4) addition of 0.3 mM ADP.

B. Representative oxygen uptake trace obtained with purified brain mitochondria in reaction buffer. Indicated with numbers: (1) Buffer alone; (2) addition of 0.42 mg mitochondria protein/ml; (3) addition of 10 mM succinate; (4) addition of 0.3 mM ADP.

C. Same as under B but with the addition of 0.2 mM L-Arg.

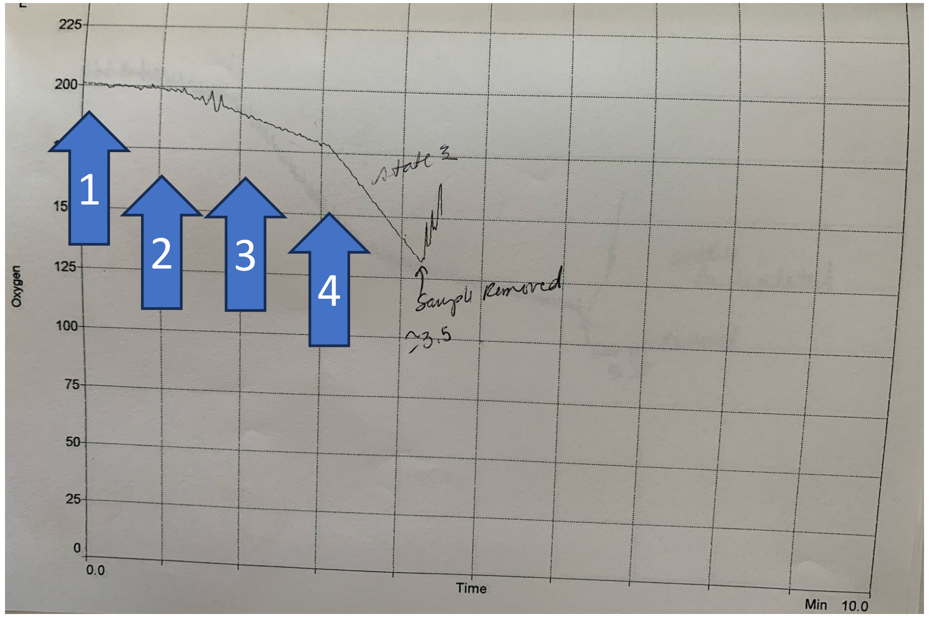

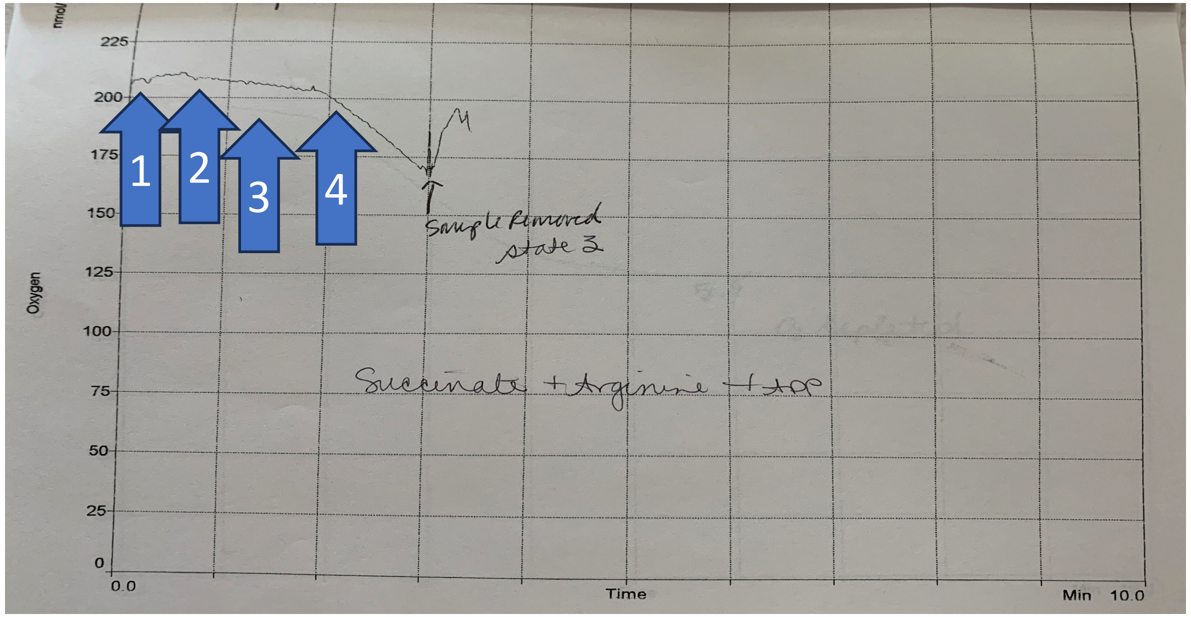

Supplementary Figure 6

PDZ domain in several NOS isoforms. Multiple sequence alignment performed with CLUSTALW

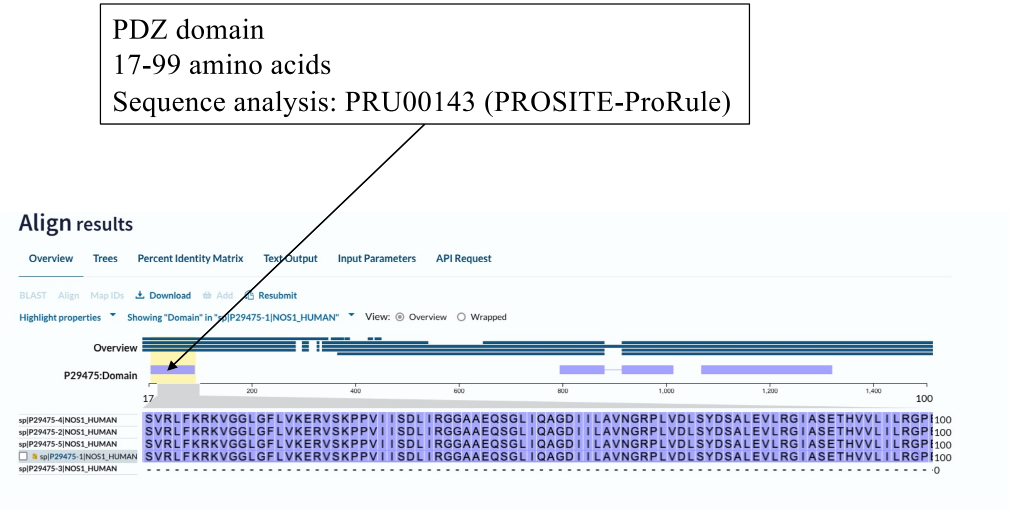

Supplement: Supplementary file 1 [file brainsci-13-01534-s001.zip › Supplementary_Figures.pdf]
